# Flexible/Bendable Acoustofluidics Based on Thin-Film Surface Acoustic Waves on Thin Aluminum Sheets

**DOI:** 10.1021/acsami.0c22576

**Published:** 2021-04-04

**Authors:** Yong Wang, Qian Zhang, Ran Tao, Jin Xie, Pep Canyelles-Pericas, Hamdi Torun, Julien Reboud, Glen McHale, Linzi E. Dodd, Xin Yang, Jingting Luo, Qiang Wu, YongQing Fu

**Affiliations:** † The State Key Laboratory of Fluid Power and Mechatronic Systems, Zhejiang University, Hangzhou 310027, China; ‡ Faculty of Engineering and Environment, University of Northumbria, Tyne NE1 8ST, U.K.; § Key Laboratory of 3D Micro/Nano Fabrication and Characterization of Zhejiang Province, School of Engineering, Westlake University, Hangzhou 310024, China; ∥ Shenzhen Key Laboratory of Advanced Thin Films and Applications, College of Physics and Optoelectronic Engineering, Shenzhen University, Shenzhen 518060, China; ⊥ Department of Integrated Devices and Systems, MESA+ Institute, University of Twente, Enschede 7522NH, The Netherlands; # Division of Biomedical Engineering, James Watt School of Engineering, University of Glasgow, Glasgow G12 8LT, U.K.; ¶ Institute for Multiscale Thermofluids, School of Engineering, University of Edinburgh, Kings Buildings, Edinburgh EH9 3FB, U.K.; ∇ Department of Electrical and Electronic Engineering, School of Engineering, Cardiff University, Cardiff CF24 3AA, U.K.

**Keywords:** acoustofluidics, flexible devices, ZnO thin films, surface acoustic waves, aluminum sheets

## Abstract

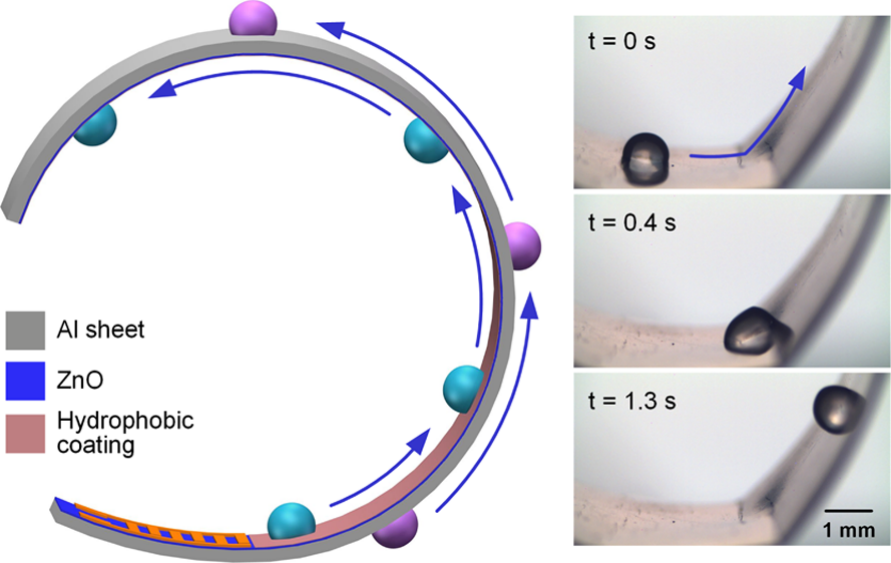

In this paper, we explore the acoustofluidic performance of zinc oxide (ZnO) thin-film surface acoustic wave (SAW) devices fabricated on flexible and bendable thin aluminum (Al) foils/sheets with thicknesses from 50 to 1500 μm. Directional transport of fluids along these flexible/bendable surfaces offers potential applications for the next generation of microfluidic systems, wearable biosensors and soft robotic control. Theoretical calculations indicate that bending under strain levels up to 3000 με causes a small frequency shift and amplitude change (<0.3%) without degrading the acoustofluidic performance. Through systematic investigation of the effects of the Al sheet thickness on the microfluidic actuation performance for the bent devices, we identify the optimum thickness range to both maintain efficient microfluidic actuation and enable significant deformation of the substrate, providing a guide to design such devices. Finally, we demonstrate efficient liquid transportation across a wide range of substrate geometries including inclined, curved, vertical, inverted, and lateral positioned surfaces using a 200 μm thick Al sheet SAW device.

## 1 Introduction

The ability to actuate liquids along flexible, deformed, three-dimensional (3D) complex surfaces is important for the design of flexible biomedical platforms for drug delivery, wearable biosensors, and lab-on-a-chip (LOC) diagnostic applications,^1−4^ as well as for smart microsystems such as those used for soft robotic control.^5,6^ They have shown superior advantages over rigid solutions such as flexibility, deformability, structure compactness, and conformability. Acoustic wave technologies, especially those based on piezoelectric thin films, have attracted great attention for microfluidic actuation and manipulation due to their advantages such as simple fabrication, remote and effective driving capability, and multifunctionality.^7−9^ Using thin-film surface acoustic wave (SAW) devices, such as those fabricated on ZnO or AlN films, various essential microfluidic functions can be achieved, including streaming,^3^ concentration,^10^ pumping,^11^ mixing,^12^ jetting, and nebulization.^13,14^

However, most thin-film acoustofluidic devices have been built on rigid substrates such as silicon and glass,^13−15^ preventing their applications to those that require flexibility, deformability, and integration within complex shapes and structures. In addition, many of these rigid substrates have limited efficiencies in enabling fluid transport on the device surface, due to the fact that forces acting on the liquid have a relatively small horizontal component (but a strong normal one). This can be measured by the Rayleigh angle (e.g., refraction angle into the liquid), θ_R_ = sin^–1^(*C*_F_/*C*_S_),^16,17^ where *C*_F_ is the sound speed in the liquid and *C*_S_ is the SAW propagation speed in the substrate. The phase velocities of Rayleigh waves on a Si substrate and a LiNbO_3_ substrate are 4680 and 3990 m/s,^18,19^ respectively, thus generating the Rayleigh angles in water of ∼22° for the LiNbO_3_-based SAW device and ∼21° for the ZnO/Si thin-film SAW device (*C*_F_ for water = 1495 m/s).^19,20^ Substrates with lower acoustic velocities (e.g., aluminum has an acoustic speed of ∼2888 m/s and generates a Rayleigh angle of ∼31.2°) are more attractive for fluid transport on their surfaces.^21^

**Figure 1:**
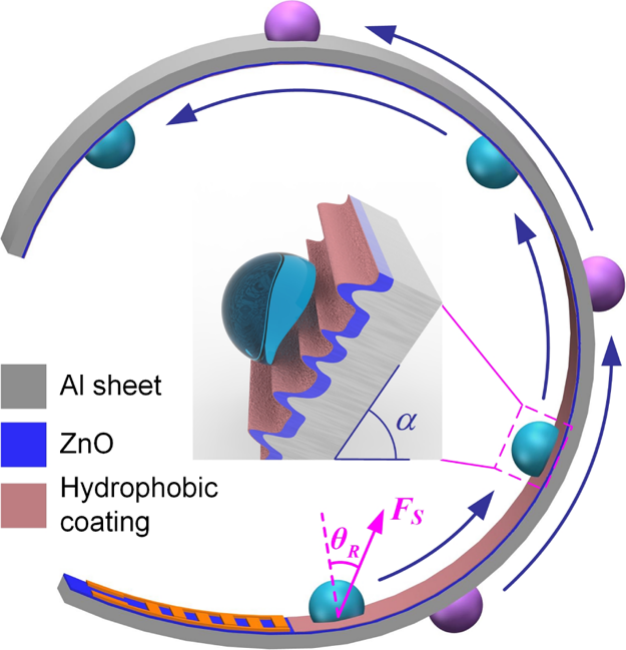
Schematic of droplet transportation along a curved surface using a flexible SAW device made by ZnO deposition on an Al sheet. A pseudo-Rayleigh or Lamb wave propagates along the sheet/foil, thereby enabling the actuation of the droplet on both sides of the SAW device. The inset shows the droplet on the hierarchically textured surface with both slippery and sticky nature for efficient droplet transportation.

ZnO films deposited on Al sheets (including thin sheets and foils) exhibit low film stress, useful film adhesion, and significantly reduced acoustic energy dissipation,^22,23^ compared to other flexible substrates, such as polymers.^24−26^ When the Al sheets are of submillimeters in thickness, they can be easily bent or deformed, and then maintain their new shapes, or be further bent to other shapes or bent back to their original shapes (see Figure 1 for a simple illustration).^27,28^ Moreover, as the Al sheets/foils are commonly fabricated with a cold-rolling manufacturing process, their surfaces have groove patterns with microscale roughness.^29^ When the ZnO thin films are deposited onto their surfaces, a nanostructured morphology forms on top of this microscale roughness (see the inset in Figure 1).^5^ If the ZnO/Al surface is further coated with a hydrophobic layer, a hierarchically textured and hydrophobic surface is generated, which is not only slippery (showing a low contact angle hysteresis and reducing the droplet pinning force) but also sticky (retaining an appropriate receding contact angle to ensure the droplet adhering to the inclined surface) for efficient liquid transportation on the deformable platform.

Thinner substrates are easier to bend, but this comes to the detrimental effect for acoustofluidic performance, as it becomes difficult to support efficient microfluidic actuation when the thickness of the Al foils is below 50 μm.^30^ This is mainly due to their poor stiffness, which causes a large deformation at a high RF power, thereby causing significant dissipation of acoustic energy into the substrate. Another major factor is the change of wave modes as the Rayleigh waves change into Lamb waves,^22^ which is less efficient than Rayleigh waves for fluidic actuation.^31^ Conversely, thick Al sheets (e.g., >1 mm) do not easily deform. We thus hypothesized that there should be an optimum thickness range (e.g., above 100 μm but below a millimeter) for efficient, yet flexible acoustofluidics.

In this paper, we aim to provide a guide for the design and manufacture of flexible, yet efficient acoustofluidic devices, by systematically investigating acoustofluidic behaviors of ZnO/Al sheet SAWs and comparing the performance with those of conventional ZnO/Si SAWs, focusing on thickness effects on wave modes and microfluidic performance. Theoretical calculations using a stiffness matrix method are presented to investigate the changes of acoustic wave velocities and amplitudes caused by the bending of the SAW devices. We demonstrate that thickness and deformability can be optimized for specific applications. In our example of Al sheets, a thickness of 200 μm enables optimal fluid transportations along various mechanically bent/deformed surfaces, paving the way for flexible/bendable or wearable applications.

## 2 Materials and Methods

ZnO films of ∼5 μm thick were deposited onto commercially available Al foil/sheet substrates (with thicknesses of 50 ± 5, 200 ± 5, 600 ± 10, and 1500 ± 10 μm) using DC magnetron sputtering processes. For the Al foils with a thickness of 50 μm, they were put on a bulk Al plate substrate to keep the flatness during the thin-film deposition. The films were also deposited onto a 4-inch silicon (100) wafer (500 μm thick) for comparisons. For the film deposition, a zinc target with a purity of 99.999% was used. ZnO films were deposited onto the above substrates using an Ar/O_2_ gas flow rate of 10/13 sccm, a DC target power of 400 W, and a chamber pressure of ∼3 mTorr. The distance between the zinc target and the sample holder was 70 mm. In addition, the sample holder was rotated to obtain uniform ZnO thin films. Crystal orientations of the deposited ZnO films were analyzed using X-ray diffraction (XRD, D5000, Siemens) with Cu Kα radiation (λ = 1.5406 Å). Surface morphologies of ZnO films were observed using a scanning electron microscope (SEM, S-4100, Hitachi). SAW devices were fabricated on the prepared substrates by patterning the Cr (20 nm)/Au (100 nm) film to form the interdigital transducer (IDT) electrodes using standard photolithography and lift-off processes. Each IDT was composed of 60 pairs of fingers, with a spatial periodicity of either 64 or 200 μm, and an acoustic aperture of 5 mm. The reflection spectra (*S*_11_) of the SAW devices were measured using an RF network analyzer (Agilent E5061B). The electromechanical coupling coefficient *k*^2^ of the SAW device was experimentally determined using the following equation derived from the Smith’s equivalent model^32−34^
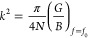
1where *N* is the number of IDT finger pairs, *G* is the conductance (real part), and *B* is the susceptance (imaginary part) of the electrical admittance *Y* = *G* + j*B*, at the central frequency, respectively. The values of *G* and *B* can be obtained from the Smith charts of the reflection coefficient (*S*_11_) at the central resonant frequency from a network analyzer.

The surfaces of the SAW devices were treated with a layer of ∼200 nm thick fluoropolymer coating (CYTOP, Asahi Glass Co., Tokyo, Japan) and heated to 120 °C for 10 min in order to make the device surface hydrophobic. A drop shape analyzer (Kruss DSA30S) was used to characterize the hysteresis resistance force of the droplet movement through measuring the advancing angle and receding angle.^35^ The measured static contact angle, advancing angle, receding angle, and contact angle hysteresis of the droplet (1 μL) on the device surface are listed in Table S1 in the Supporting Information. After the hydrophobic treatment, the contact angle hysteresis of the droplet (1 μL) on the ZnO/Si surface was decreased from 62.9 ± 8 to 27.2 ± 6°. However, for the surface of ZnO/Al plate (1500 μm thick), the contact angle hysteresis was decreased from 25.1 ± 10 to 13.1 ± 5°.

For microfluidic testing, the SAW devices were placed on top of an aluminum alloy test holder to minimize acoustic heating.^36^ An RF input signal was generated using a signal generator (Marconi 2024) and amplified using a power amplifier (Amplifier research, 75A250) before being fed into the input IDTs. The input SAW power was measured using an RF power meter (Racal Instruments 9104). The microfluidic behaviors (including pumping and jetting) were observed using a standard video camera (60 fps) and a high-speed video camera (FASTCAM-ultima APX with a frame rate of 40,000 fps). A photograph of the experimental setup for the microfluidic test is shown in Figure S1 in the Supporting Information.

To understand the wave vibration patterns on Al sheets with different thicknesses, finite element analysis (FEA) was performed using COMSOL Multiphysics (5.3a) with solid mechanics and electrostatics modules. A simplified two-dimensional (2D) model with ideal material parameters, one pair of IDT electrode, and periodic boundary conditions were used to simulate the wave vibration patterns on different Al sheets. Moreover, a modified stiffness matrix method and elasto-plastic theory were implemented in MATLAB to analyze the frequency shifts and amplitude changes of the SAW device under different bending strains.^37,38^ To simplify the calculation process, a pure bending condition, a zero residual stress, and no cracks were assumed after the bending. The modeling and calculation details can be seen in the Methodology in the Supporting Information.

## 3 Results and Discussion

### 3.1 Film and Device Characterization

**Figure 2:**
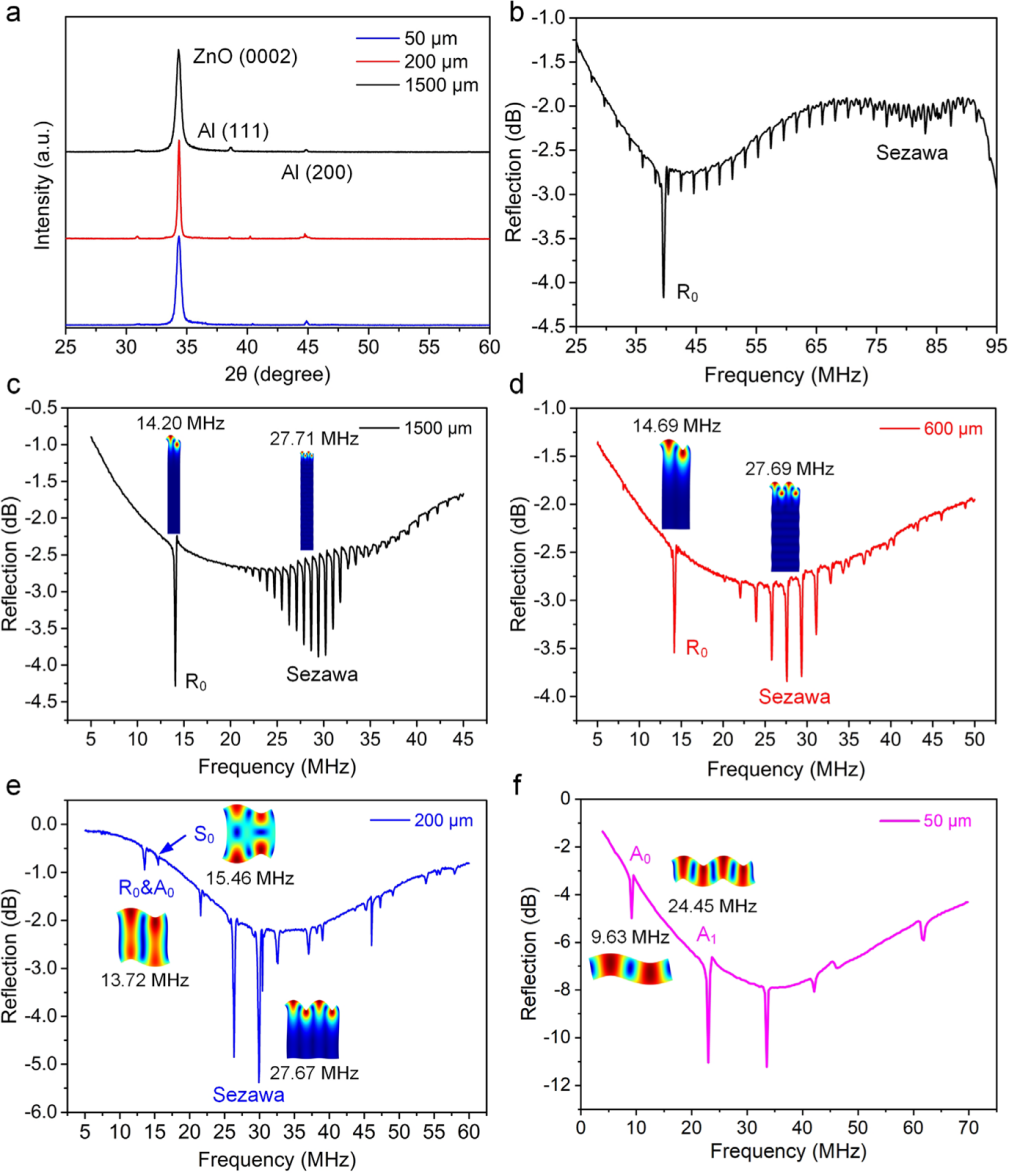
(a) XRD patterns of ZnO thin films on Al sheet substrates with various thicknesses. (b) Signal reflection spectra (*S*_11_) of the ZnO/Al plate (1500 μm thick) SAW device at a wavelength of 64 μm. FEA simulation and experimental verification of wave vibration modes and their corresponding resonant frequencies for the SAW devices with 200 μm wavelength and varied Al sheet thicknesses of (c) 1500, (d) 600, (e) 200, and (f) 50 μm.

Figure 2a shows the XRD spectra of ZnO thin films on Al substrates with different thicknesses. The results show that all the ZnO films on Al foils, Al sheets, and Al plates have a dominant diffraction peak at 2θ of ∼34.3°, indicating a preferential growth orientation along the *c*-axis (0002).^23^ Figure S2 in the Supporting Information shows the cross-sectional SEM image of the ZnO films on Al foils, indicating the formation of a columnar morphology of the ZnO microstructure. The ZnO films deposited on the Si substrate, which are used for comparison, also show a good *c*-axis (0002) orientation (see Figure S3 in the Supporting Information). Figure 2b shows the reflection spectra (*S*_11_) of the ZnO/Al plate (1500 μm thick) SAW device with a wavelength of 64 μm. For comparison, a ZnO/Si SAW device with the same electrode configuration was fabricated, with the reflection spectra (*S*_11_) shown in Figure S4 in the Supporting Information. The SAW propagation speed in the ZnO thin film (2700 m/s) is smaller than those on the Al plate (∼2888 m/s) and Si (4680 m/s) substrates, thus generating both the Rayleigh mode (R_0_) and Sezawa mode.^17,18^

Figure 2c–f shows the effects of the Al sheet thickness (from 50 to 1500 μm) on wave modes when the device wavelength is 200 μm. The thickness/wavelength ratio plays an important role in wave mode selection. When this ratio is much larger than one, Rayleigh waves are dominant. On the contrary, if the ratio is smaller than one, Lamb waves are dominant, while hybrid modes (both Rayleigh and Lamb waves) are commonly observed for a ratio near one.^22^ The FEA simulation results for wave vibration patterns on different Al sheets show that when the Al sheet thickness (i.e., 600 and 1500 μm) is larger than the device wavelength, the SAW devices generate a Rayleigh mode and a Sezawa mode, as depicted in Figure 2c,d. When the Al sheet thickness is in a similar range to the device wavelength, the A_0_ mode and pseudo-Rayleigh mode are hybridized together at a frequency of 13.72 MHz, and the pseudo-S_0_ mode and the Sezawa mode are also obtained, as illustrated in Figure 2e. When the Al sheet thickness is further decreased to 50 μm, the wave vibration modes are changed into the typical Lamb waves, without Rayleigh and Sezawa modes observed, as shown in Figure 2f. The simulated wave modes for SAW devices with the wavelengths of 64 μm and 200 μm and Al sheet thicknesses from 50 to 1500 μm are shown in Figure S5 in the Supporting Information.

### 3.2 Bending Effects on Device’s Frequency and Amplitude

For flexible acoustofluidic applications, the SAW devices are often bent into different shapes. Therefore, we further studied the effects of bending on device’s resonant frequency and acoustic wave amplitude. Here, a modified stiffness matrix method was used for modeling and calculations.^37^ For the modeling, the Al sheet thickness is set as 200 μm, and the thickness of the ZnO thin film is 5 μm. A mechanical strain *st*/2*r* is defined as the strain on the ZnO surface, where *t* is the thickness of the device and *r* is the curvature radius. For a tensile strain, *s* is selected as 1, and for a compressive strain, *s* is −1. When the SAW device is bent, all the densities and elastic constants of ZnO and Al as well as the device wavelengths will change. The total frequency shifts can be regarded as the sum of several frequency shift components caused by the changes of the density, elastic constant, device wavelength, and the stress, respectively.^39^

**Figure 3:**
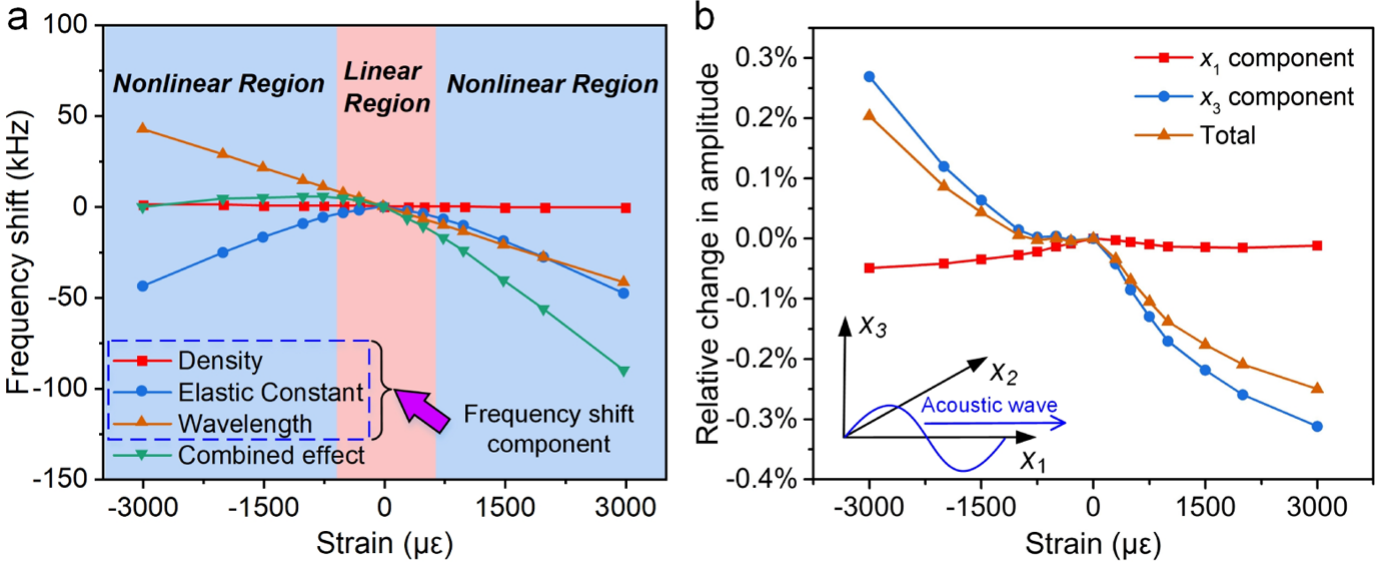
(a) Calculated contribution components of density, elastic constant, and device wavelength changes under different bending strains to device’s frequency shifts (e.g., the A_0_ mode ). (b) Theoretically calculated relative acoustic wave amplitude changes (e.g., the A_0_ mode) on the top surface of a 200 μm thick Al sheet device under different bending strains. The *x*_1_ and *x*_3_ components are the wave vibration components in the *x*_1_ (along the wave propagation) and *x*_3_ (perpendicular to the substrate surface) directions.

Figure 3a shows the calculated frequency shifts (A_0_ mode) due to the changes of density, elastic constant, and device’s wavelength as a function of bending strains. The frequency shift caused by the density change is relatively small (<1 kHz under strain levels of 3000 με), while the change in the elastic constant has a larger impact than that of the wavelength, leading to an apparent nonlinear effect with the increase of strain. Nevertheless, for flexible acoustofluidic testing, a bending curvature of 50–100 m^–1^ can be considered for most applications, which leads to calculated strains of −2750 to −1375 με, corresponding to frequency shifts of about 1.2 to 5.1 kHz. Here, the positive strain represents the bending outward, while the negative strain is generated by the bending inward. With such a small frequency shift, the acoustic wave speed change is smaller than 0.3%. Before each acoustofluidic test, we have bent the SAW device into a new shape and maintained this shape, and its resonant frequency was measured using a network analyzer. Then, the measured frequency value was input into the signal generator to excite the acoustic wave for microfluidic actuation.

As the acoustic wave amplitude determines the SAW driving force, the relative changes in acoustic wave amplitude (A_0_ mode) after the bending were further calculated, and the obtained results are shown in Figure 3b. Here, direction *x*_1_ is defined as that along the acoustic wave propagation direction (longitudinal component), while direction *x*_3_ is defined as that perpendicular to the substrate surface (transverse or shear vertical component).^40^ When a positive strain is applied to the SAW device, the amplitudes of *x*_1_ and *x*_3_ components both decrease with the strain. With the application of a negative strain, the *x*_1_ component decreases while the *x*_3_ component increases with the increase of strain values. For the bending strains considered previously (from −2750 to −1375 με, or curvature from 50 to 100 m^–1^), the relative changes in acoustic wave amplitude are less than 0.3%, indicating that the bending only has a limited influence on the acoustic wave amplitude. We have also demonstrated in experiment that when the thin Al sheet substrate (200 μm thick) is bent to nearly 90° with a curvature of ∼50 m^–1^, in the pseudo-Rayleigh or A_0_ mode, the SAW device still carries a strong signal (see Figure S6 in the Supporting Information), thereby enabling an efficient microfluidic actuation on the flexible surfaces.

### 3.3 Acoustofluidic Demonstration Using the ZnO/Al Plate SAW Device

As a baseline, we first chose the 1500 μm thick Al plate SAW device and investigated its microfluidic actuation behaviors along flat and inclined surfaces and compared them with those of well-studied and conventional ZnO/Si SAW devices.^41^ Because the device wavelength has a significant effect on microfluidic performance, for these comparisons, we chose the SAW devices with the same wavelength of 64 μm. When a droplet is placed on the surface and maintained horizontally, the droplet movement on the Al plate surface is a combination of rolling and sliding (see the captured images shown in Figure S7a). Whereas for the ZnO/Si SAW device, the droplet movement on the surface is dominated by sliding and jumping (see the pumping images shown in Figure S7b). This is due to the lower acoustic velocity of the ZnO/Al plate SAWs compared to ZnO/Si SAWs, thereby generating a larger Rayleigh angle (31.2° compared with 20.9°), resulting in a larger horizontal component of the SAW driving force and consequently a more significant horizontal deformation (as evidenced in Figure S7).

**Figure 4:**
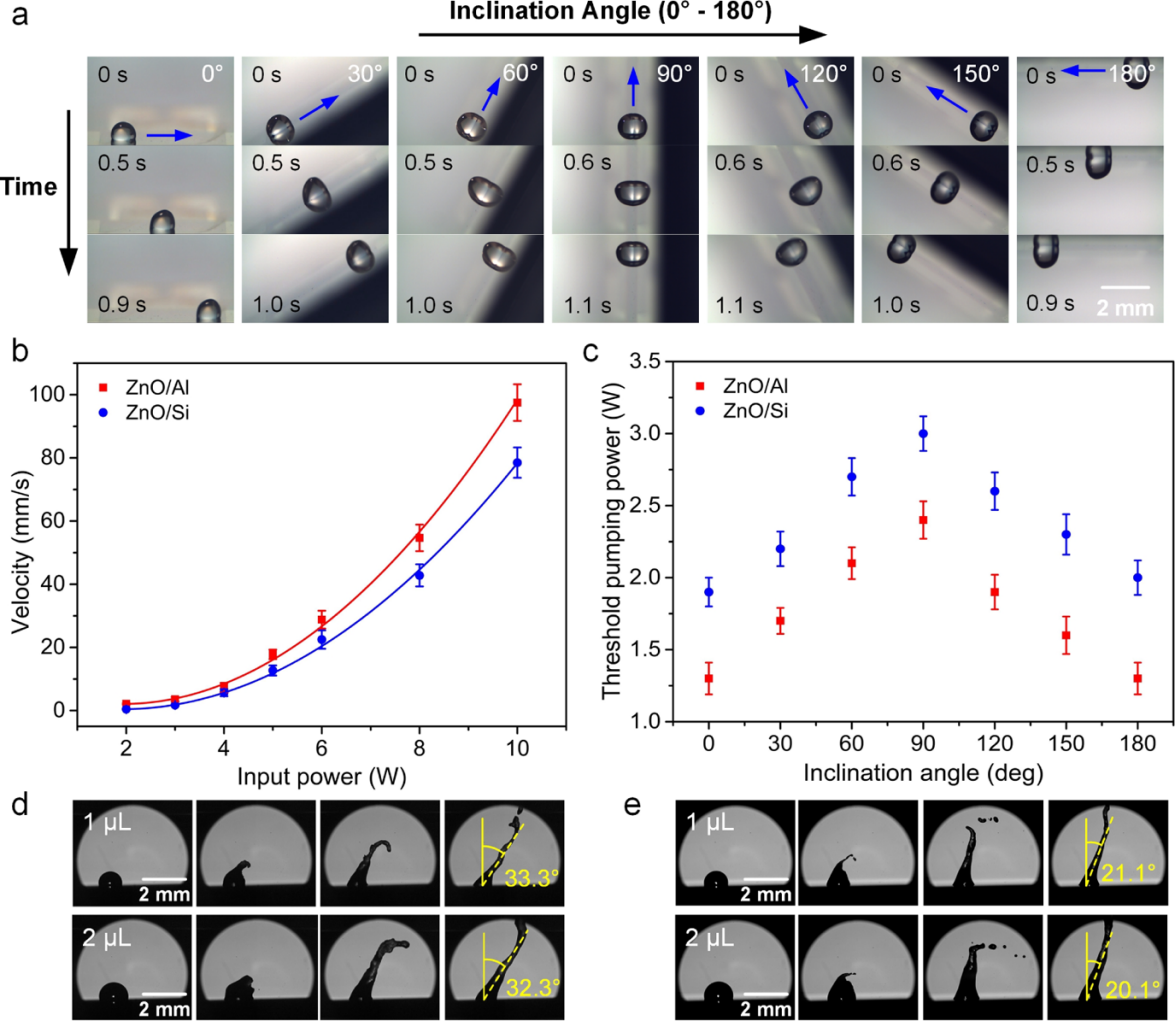
(a) Droplet (1 μL) pumping images along inclined surfaces with inclination angles from 0 to 180° using the ZnO/Al plate SAW device with an input power of 5 W. (b) Comparisons of average pumping velocities for the droplet on the flat surface under different input powers between the ZnO/Al plate SAW device and the ZnO/Si SAW device. (c) Threshold powers of pumping a 1 μL droplet for the ZnO/Al plate SAW device and the ZnO/Si SAW device at different inclination angles. High-speed images indicating the jetting angles using the (d) ZnO/Al plate SAW device and (e) ZnO/Si SAW device with the same input power of 18 W.

We further investigated microfluidic pumping characteristics when the surface is inclined with an angle α (defined as the angle at which the device substrate was tilted along the horizontal plane, as shown in Figure 1). Here, the droplet size and gravity play important roles, and when the droplet size is above a certain value, it slides down (Movie S1) or drops from the device surface.^42,43^ This threshold decreased with increased inclination angle when the inclination angle is smaller than 90°, and increased with inclination angles increasing from 90 to 180°, as shown in Figure S8 in the Supporting Information. We have found that at an inclination angle of 90° (i.e., vertical alignment), the maximum droplet volume that can be pumped uphill shows its smallest value (∼3 μL) compared with those at the other inclination angles, because the gravity component along the inclined surface reaches the maximum value. Figure 4a demonstrates that when the droplet volume is 1 μL, it can be efficiently pumped along arbitrary inclined surfaces.

Figure 4b,c compares the pumping performance between the ZnO/Al plate SAW device and the ZnO/Si SAW device with the same wavelength of 64 μm. Clearly, for both these SAW devices, the average pumping velocity for the droplet on the horizontal surface is increased with the increase of input power. At the same power, the droplet average pumping velocity using the ZnO/Al plate device is larger than that of the ZnO/Si SAW device, as shown in Figure 4b. Besides, the ZnO/Al plate SAW device also requires a lower threshold power for microfluidic actuation when compared to that of the ZnO/Si SAW device at the same inclination angle (e.g., ∼1.3 W for the ZnO/Al plate SAWs and ∼2.0 W for the ZnO/Si SAWs for the horizontal surface). This threshold actuation/pumping power is defined as the minimum power to initiate the droplet movement, and a lower threshold power means that a smaller input power is needed to actuate the droplet. Therefore, in comparison with the ZnO/Si SAW device, the ZnO/Al plate SAW device provides a better microfluidic actuation performance.

In addition to the increased Rayleigh angle as previously mentioned, the enhanced microfluidic actuation performance can also be attributed to a higher electromechanical coupling coefficient of the ZnO/Al plate (1.57%) SAWs than that of ZnO/Si SAWs (1.08%), which enables more applied power to be transformed into acoustic energy given the same input power, thereby improving the energy efficiency. Moreover, the formation of a hierarchically textured surface on Al substrates generates a lower contact angle hysteresis, which then reduces the threshold actuation power.^5^

The Rayleigh angles can be further confirmed when performing jetting of the droplets at higher powers (see Figure S9 in the Supporting Information). After about 4 ms of actuation, a coherent liquid beam became dominant. The droplet jetting angles were measured as 33° for the ZnO/Al plate SAW device and 21° for the ZnO/Si SAW device, approximately following the Rayleigh angles of the corresponding SAW devices, as shown in Figure 4d,e.

### 3.4 Optimization of Al Sheet Thickness for Acoustofluidics

To provide a guide for the design of flexible acoustofluidic devices, we systematically investigated the effects of the Al sheet thickness on microfluidic actuation performance, using a SAW device with a wavelength of 200 μm. Table 1 summarizes threshold powers for pumping/jetting of a 1 μL droplet for the SAW devices with different Al sheet thicknesses using different wave modes. Results show that for the 50 μm thick Al foil SAW device, much higher powers are needed to transport the droplet compared to those of thicker Al substrate SAWs. The pumping performance of the A_0_ mode is better than that of the S_0_ mode and no droplet jetting was observed for any of the Lamb waves. The three types of thicker Al sheet SAW devices show comparable threshold pumping and jetting powers when using either Rayleigh or hybrid modes. No droplet jetting was observed when using Sezawa modes, mainly due to the fact that these are guided waves, which propagate along the interface between the piezoelectric layer and the substrate, thus dissipate less energy into surface droplets. In addition, the droplet jetting phenomena using Lamb wave modes (e.g., A_0_ or S_0_ modes) of 200 μm thick Al sheet SAW device have been observed (Movies S2 and S3).

**Table 1:** Threshold Powers for Pumping/Jetting of a 1 μL Droplet for the SAW Devices on Al Sheets of Different Thicknesses at the Same Wavelength of 200 μm Using Different Wave Modes

Al sheet thickness (μm)		50	200	600	1500
threshold pumping power (W)	R_0_		0.5	0.6	0.5
	A_0_	2.6	0.5		
	S_0_	7.0	2.1		
	Sezawa		16	14	16
threshold jetting power (W)	R_0_		16	18	16
	A_0_		16		
	S_0_		22		
	Sezawa				

**Figure 5:**
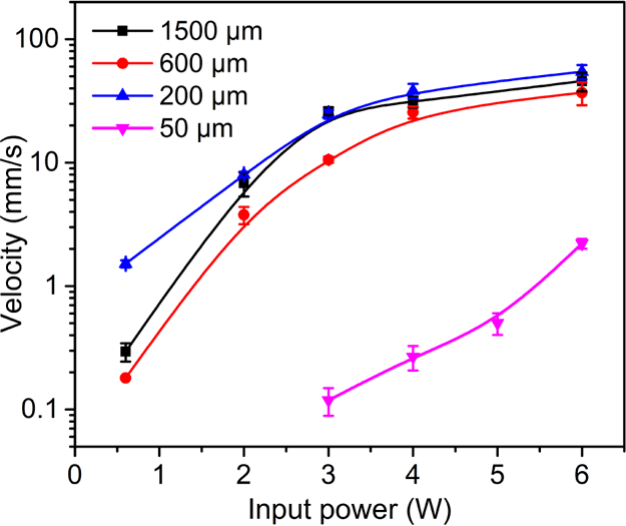
Droplet (1 μL) average pumping velocities as a function of input power, for thick Al plate (i.e., 600 and 1500 μm) SAW devices driven using the Rayleigh mode and for thin Al sheet (200 μm thick) and Al foil (50 μm thick) SAW devices driven using the pseudo-R_0_ or A_0_ mode.

Figure 5 shows the average pumping velocities of a 1 μL droplet for the SAW devices with different Al sheet thicknesses using the Rayleigh mode or the A_0_ mode with input powers varying from 0.6 to 6 W. At the same input power, the 50 μm thick Al foil SAW device shows the lowest droplet pumping velocity, consistent with its flexural wave mode, which does not transfer much energy into the liquid. The other reasons include the large deformation of the substrate (Movies S4 and S5), the increased acoustic dissipation, thus the reduced microfluidic driving efficiency. Therefore, although the Al foil-based SAW devices have relatively good flexibility, they do not exhibit the best microfluidic actuation performance. The 200 μm thick Al sheet SAW device supports hybrid modes (e.g., pseudo-Rayleigh and Lamb waves at the same frequency, Figure 2e) with comparable pumping performance to those of 600 μm and 1500 μm thick Al plate SAW devices using the Rayleigh mode, making them highly suitable for application of flexible/bendable acoustofluidics. In addition, the increase of the ZnO thin-film thickness might further enhance the microfluidic actuation performance on these flexible surfaces, due to its piezoelectric nature.

### 3.5 Demonstration of Acoustofluidics on Flexible/Bendable Substrates: the 200 μm Case

**Figure 6:**
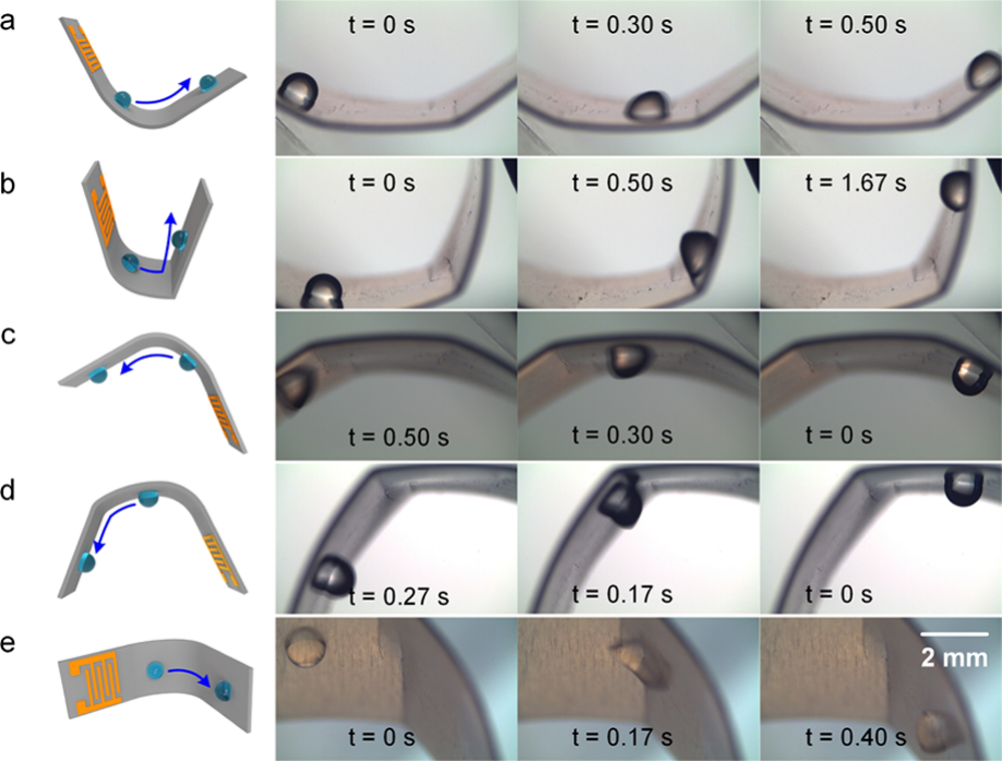
Demonstration of droplet pumping/transportation using ZnO thin-film SAWs on the bent Al sheet (200 μm thick) substrates. (a,b) 1 μL droplet transportation along different bending curvature surfaces. (c,d) 1 μL droplet transportation along different spatial position surfaces (e.g., inverted and downward). (e) 1 μL droplet transportation along the laterally bent surface. The input SAW power is 16 W.

To demonstrate the range of applications that could be achieved with the optimum 200 μm thick Al sheet SAW device, it was deformed into different shapes and tilted to different orientations. Figure 6a,b shows that the droplet can be efficiently transported on the bent surfaces with curvatures varying from 50 to 100 m^–1^ (see Movie S6), which corresponds to a nearly “U”-shaped path. We have also tilted the bent SAW device to different angles and demonstrated efficient transportation of the droplet on different spatial positions (e.g., inverted and downward), as shown in Figure 6c,d. For the inverted surface, the maximum droplet volume that can be pumped is about 7 μL (Movie S7), otherwise the droplet would drop down from the surface. Figure 6e demonstrates the pumping/transportation of the droplet along the laterally bent surface. Therefore, in theory, when the droplet size is smaller than a certain value (here ≤3 μL), they can be efficiently transported on arbitrarily shaped and positioned surfaces using this SAW device (including on the backside, Movie S8, although at a slower speed). Otherwise, gravity of the droplet will become dominantly influenced. Furthermore, during the entire bending process, no distinct deterioration of the microfluidic actuation performance was observed, indicating great potential of thin Al sheet SAWs for flexible/bendable acoustofluidics.

## 4 Conclusions

In summary, through both systematic numerical and experimental characterization of the acoustofluidic behaviors of ZnO thin-film SAW devices fabricated on Al sheets of different thicknesses, we provide the evidence of optimum conditions for acoustofluidic functionalities on bendable and deformable surfaces. We have demonstrated a better microfluidic actuation performance of the ZnO/Al plate SAW device when compared to that of the conventional ZnO/Si SAW device and verified the efficient transportation of droplets along various inclined surfaces using these SAW devices. As the thickness is decreased, the wave vibration modes changed from Rayleigh to hybrid modes and subsequently to Lamb waves. We have shown that in this specific study, 200 μm is the optimal thickness to combine deformability and acoustofluidic actuation, enabling fluidic functions to be performed on complex 3D shapes. Our work proposes a new platform for flexible Al sheet SAW devices to perform versatile microfluidic, sensing, and diagnostic applications.

## Supporting Information Available

10 μL droplet transport on a 30° inclined surface using the ZnO/Al plate SAW device with an actuation frequency of 39.63 MHz and an input power of 7 W (AVI)
1 μL droplet jetting using the pseudo-Rayleigh or A_0_ mode of the 200 μm thick Al sheet SAW device with an actuation frequency of 13.58 MHz and an input power of 18 W (AVI)
1 μL droplet jetting using the S_0_ mode of the 200 μm thick Al sheet SAW device with an actuation frequency of 15.56 MHz and an input power of 24 W (AVI)
1 μL droplet movement on the Al foil substrate driven using the A_0_ mode of the 50 μm-thick Al foil SAW device with an actuation frequency of 9.38 MHz and an input power of 6 W (AVI)
1 μL droplet movement on the Al foil substrate driven using the S_0_ mode of the 50 μm-thick Al foil SAW device with an actuation frequency of 25.1 MHz and an input power of 6 W (AVI)
1 μL droplet transport on the bent surface using the pseudo-Rayleigh or A_0_ mode of the 200 μm thick Al sheet SAWs with an actuation frequency of 13.57 MHz and an input power of 16 W (AVI)
6 μL droplet transport on the inverted surface using the pseudo-Rayleigh mode of the 200 μm thick Al sheet SAWs with an actuation frequency of 13.57 MHz and an input power of 16 W (AVI)
1 μL droplet transport on the backside of the 200 μm thick Al sheet SAW device using the pseudo-Rayleigh mode with an input power of 5 W (AVI)
Contact angle hysteresis measurements on different device surfaces; photograph of the experimental setup for the microfluidic test; cross-sectional SEM image of the ZnO films on Al foils; XRD patterns of ZnO films on the silicon substrate; reflection spectra of the ZnO/Si SAW device; FEA simulations of wave vibration modes for the SAW devices with different Al sheet thicknesses; reflection spectra of the thin Al sheet (200 μm thick) SAW device before and after bending; high-speed droplet pumping images using the ZnO/Al plate and ZnO/Si SAW devices; maximum droplet pumping volume at different inclination angles, high-speed droplet jetting images using the ZnO/Al plate and ZnO/Si SAW devices; and methodology: model; assumption; and equation (PDF)

## Supplementary Material

am0c22576_si_001.avi

am0c22576_si_002.avi

am0c22576_si_003.avi

am0c22576_si_004.avi

am0c22576_si_005.avi

am0c22576_si_006.avi

am0c22576_si_007.avi

am0c22576_si_008.avi
