# Supplementary material for: Flexible/Bendable Acoustofluidics Based on Thin-Film Surface Acoustic Waves on Thin Aluminum Sheets
Source: ACS Appl Mater Interfaces. 2021 Apr 4;13(14):16978–86. doi: 10.1021/acsami.0c22576 (PMC8153544; doi:10.1021/acsami.0c22576)
Supplement: Supplementary file 9 — am0c22576_si_009.pdf [file am0c22576_si_009.pdf]

Supporting Information

Flexible/Bendable Acoustofluidics Based on Thin Film Surface Acoustic Waves on Thin Aluminum Sheets

Yong Wang[a,b,c,†], Qian Zhang[a,b,†], Ran Tao[d,b], Jin Xie[a,*], Pep Canyelles-Pericas[e], Hamdi Torun[b], Julien Reboud[f], Glen McHale[g], Linzi E. Dodd[b], Xin Yang[h], Jingting Luo[d], Qiang Wu[b], and YongQing Fu[b,a,*]

[a] The State Key Laboratory of Fluid Power and Mechatronic Systems, Zhejiang University, Hangzhou 310027, China

[b] Faculty of Engineering and Environment, University of Northumbria, Newcastle upon Tyne NE1 8ST, UK

[c] Key Laboratory of 3D Micro/Nano Fabrication and Characterization of Zhejiang Province, School of Engineering, Westlake University, Hangzhou 310024, China

[d] Shenzhen Key Laboratory of Advanced Thin Films and Applications, College of Physics and Optoelectronic Engineering, Shenzhen University 518060, China

[e] Department of Integrated Devices and Systems, MESA+ Institute, University of Twente, Enschede 7522NH, The Netherlands

[f] Division of Biomedical Engineering, James Watt School of Engineering, University of Glasgow, Glasgow G12 8LT, UK

[g] Institute for Multiscale Thermofluids, School of Engineering, University of Edinburgh, Kings Buildings, Edinburgh EH9 3FB, UK

[h] Department of Electrical and Electronic Engineering, School of Engineering, Cardiff University, Cardiff CF24 3AA, UK

[†] These authors contributed equally to this paper.

* Corresponding authors: xiejin@zju.edu.cn; richard.fu@northumbria.ac.uk

Table

Table S1. Static contact angle, advancing contact angle, receding contact angle and contact angle hysteresis of the droplet (1 μL) on different surfaces.

Surface type	θ_s	θ_{adv}	θ_{rec}	$\Delta\theta = \theta_{adv} - \theta_{rec}$
ZnO/Al	98.6º±1º	102.5º±3º	77.4º±7º	25.1º±10º
CYTOP/ZnO/Al	109.3º±2º	112.7º±2º	99.6º±3º	13.1º±5º
ZnO/Si	86.6º±3º	90.4º±4º	27.5º±4º	62.9º±8º
CYTOP/ZnO/Si	121.5º±2º	122.8º±2º	95.6º±4º	27.2º±6º

Figure

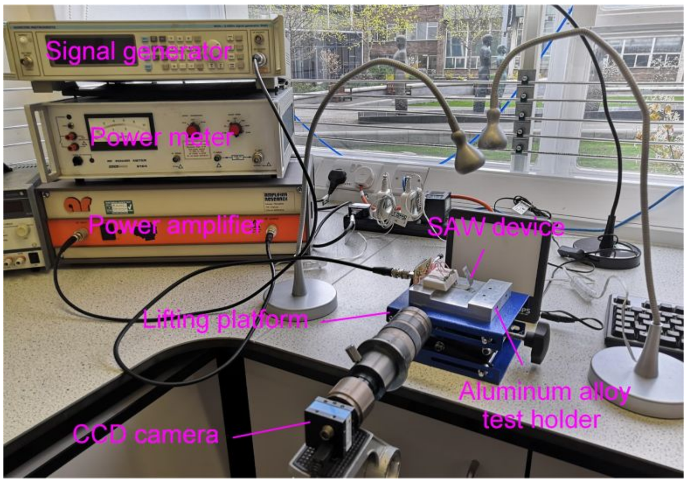

Figure S1. The photograph of experimental setup for microfluidic test.

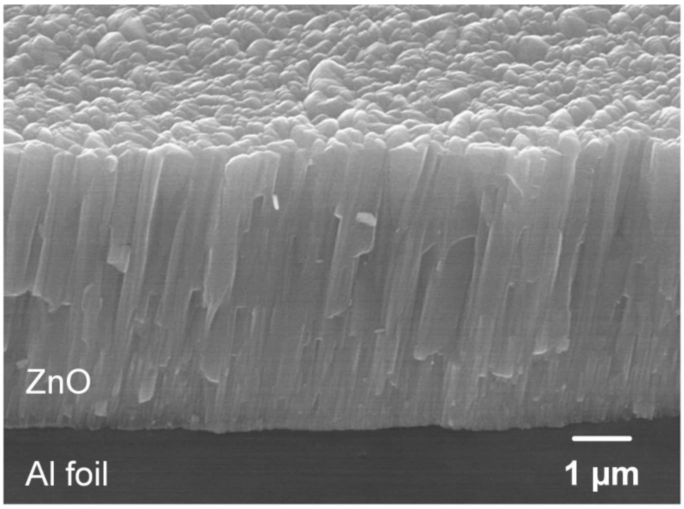

Figure S2. Cross-section SEM image of the ZnO films on Al foils, indicating a columnar morphology of ZnO microstructure.

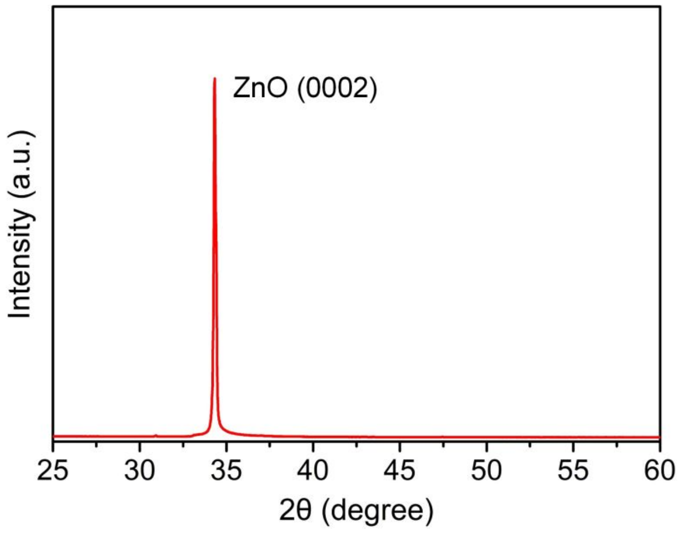

Figure S3. XRD pattern of ZnO thin films on Si substrate.

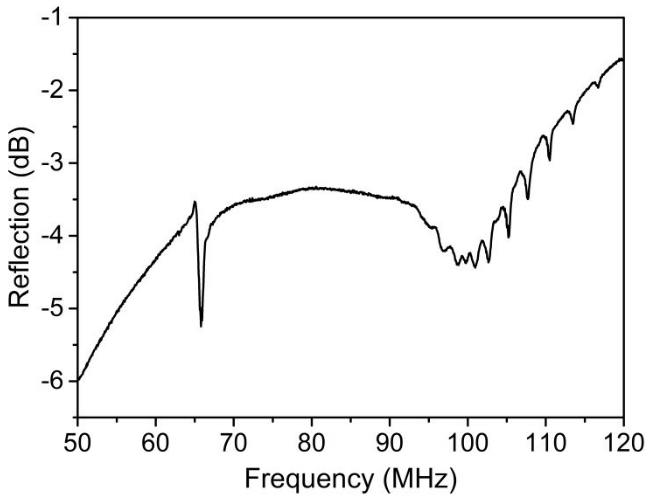

Figure S4. Signal reflection spectra (S_{11}) of ZnO/Si SAW device with the wavelength of 64 μm.

Wavelength (μm)		64				200			
Thickness (μm)		50	200	600	1500	50	200	600	1500
Mode	R0	—	42.60 MHz	42.63 MHz	42.88 MHz	—	—	14.33 MHz	14.33 MHz
	A0	41.12 MHz	—	—	—	9.72 MHz	13.84 MHz	—	—
	S0	49.73 MHz	—	—	—	25.75 MHz	15.60 MHz	—	—
	A1	—	—	—	—	24.69 MHz	—	—	—
	S1	—	—	—	—	39.75 MHz	—	—	—
	Sezawa	80.74 MHz	80.75 MHz	80.84 MHz	81.19 MHz	—	28.00 MHz	28.02 MHz	28.11 MHz

Figure S5. Finite element analysis (FEA) simulations of wave vibration modes for the SAW devices with the wavelengths of 64 μm and 200 μm and varied Al sheet thicknesses from 50 μm to 1500 μm.

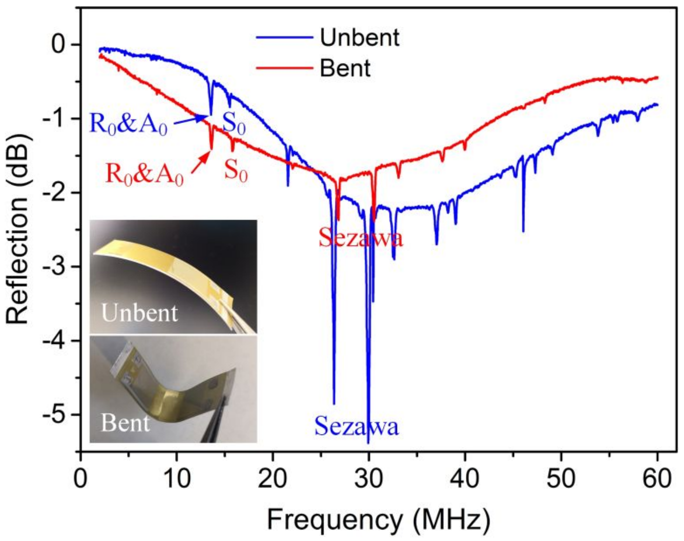

Figure S6. Reflection spectra (S_{11}) changes of thin Al sheet (200 μm thick) SAW device before and after the bending.

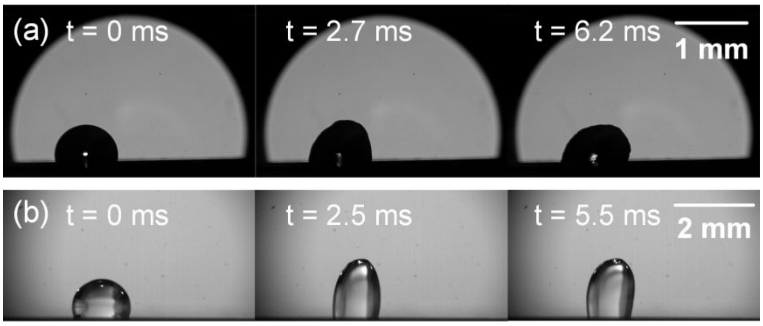

Figure S7. High-speed pumping images of the droplet (a) using ZnO/Al plate (1500 μm thick) SAW device and (b) ZnO/Si (500 μm thick) SAW device with the same wavelength of 64 μm.

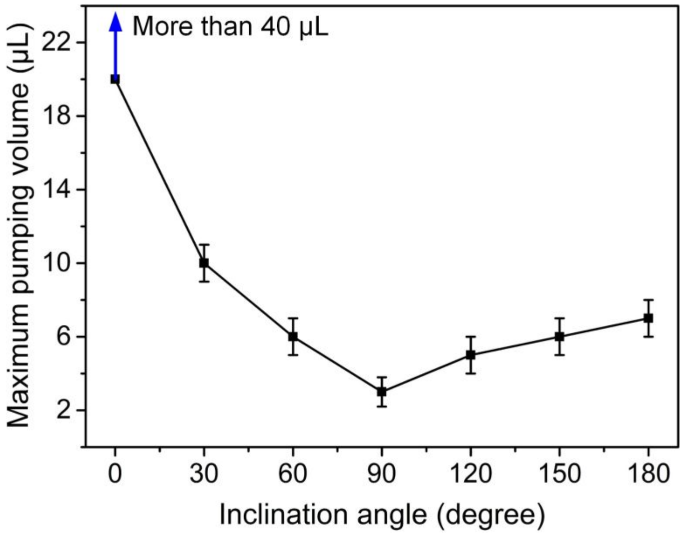

Figure S8. The maximum pumping volume of the droplet under different inclination angles.

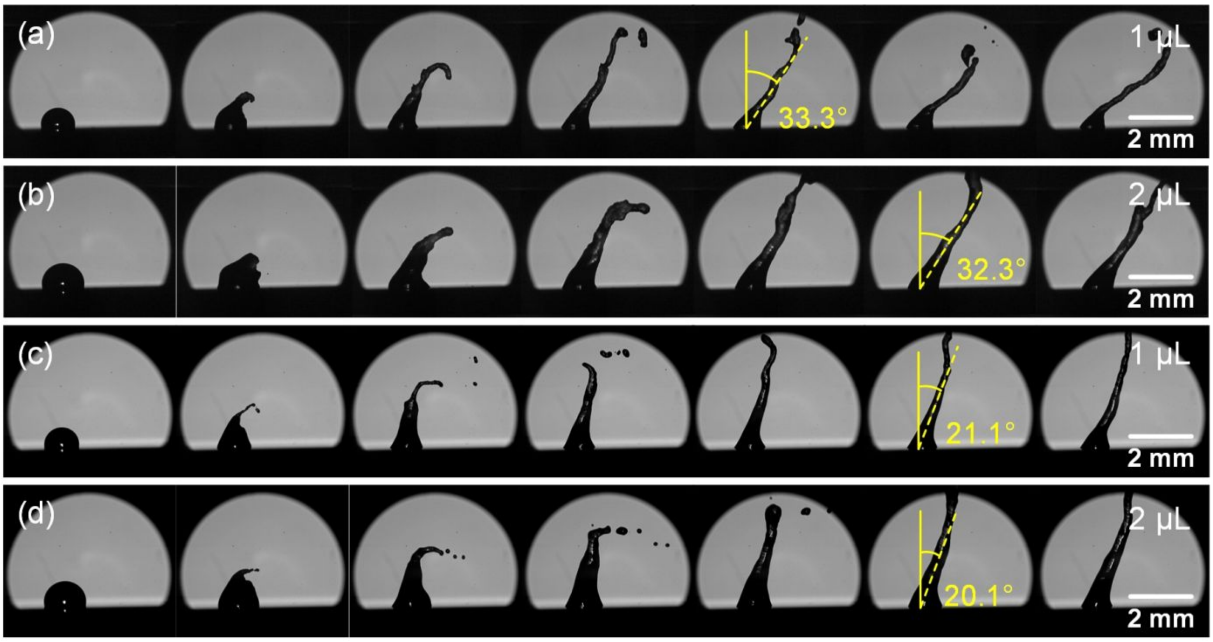

Figure S9. High-speed jetting images (1 ms per frame) of the droplet driving by the traveling wave (from left to right) using (a-b) ZnO/Al plate (1.5 mm thick) SAW device and (c-d) ZnO/Si SAW device with the same input power of 18 W.

Movie

Movie S1. 10 μL droplet transport (30 frames/s) on 30° inclined surface using the ZnO/Al plate (1.5 mm thick) SAW device with an actuation frequency of 39.63 MHz and an input power of 7 W.

Movie S2. 1 μL droplet jetting (60 frames/s) using pseudo-Rayleigh or A_0 mode of 200 μm thick Al sheet SAW device with an actuation frequency of 13.58 MHz and an input power of 18 W.

Movie S3. 1 μL droplet jetting (60 frames/s) using S_0 mode of 200 μm thick Al sheet SAW device with an actuation frequency of 15.56 MHz and an input power of 24 W.

Movie S4. 1 μL droplet movement (30 frames/s) on the Al foil substrate driving using A_0 mode of 50 μm-thick Al foil SAW device with an actuation frequency of 9.38 MHz and an input power of 6 W.

Movie S5. 1 μL droplet movement (30 frames/s) on the Al foil substrate driving using S_0 mode of 50 μm-thick Al foil SAW device with an actuation frequency of 25.1 MHz and an input power of 6 W.

Movie S6. 1 μL droplet transport (25 frames/s) on the bent surface using the pseudo-Rayleigh or A0 mode of 200 μm thick Al sheet SAWs with an actuation frequency 13.57 MHz and an input power of 16 W.

Movie S7. 6 μL droplet transport (25 frames/s) on the inverted surface using the pseudo-Rayleigh mode of 200 μm thick Al sheet SAWs with an actuation frequency 13.57 MHz and an input power of 16 W.

Movie S8. 1 μL droplet transport (25 frames/s) on the backside of the 200 μm thick Al sheet SAW device using the pseudo-Rayleigh mode with an input power of 5 W.

Methodology

Model. The model used for modeling the bending effects is based on a stiffness matrix method,[1] which is suitable for the analysis of plane acoustic waves propagating in multilayered media. Each layer of the medium corresponds to its own stiffness matrix. The global stiffness matrix of the whole multilayered media can be calculated using a recurrence equation, as presented in Eq. (7). Applied with the boundary condition, the resonant frequency and vibration amplitude of acoustic wave can be solved. The stiffness matrix of a single-layer medium is an 8-order matrix obtained from the constitutive equations and wave equations (see Eqs. (1) to (5)), where the material properties and strain distributions need to be constant. However, the material parameters (e.g., density and elastic constant) are different in different layers due to the nonuniform strain distributions. Therefore, the ZnO and Al layers are further divided into 50 sublayers to make the strain distributions in each sublayer approximately be uniform.

Assumption. The bending of the SAW device is assumed to be a pure bending, under which all the densities and elastic constants of ZnO and Al as well as the device's wavelength are changed. The total frequency shifts can be regarded as the sum of several frequency shift components caused by the changes of the density, elastic constant, device's wavelength and the stress, respectively. For example, when calculating the density component, the initial density distribution is replaced with the density distribution under bending, and the corresponding frequency shift is obtained. Considering the applications in this work, no residual stress is taken for the calculation. Therefore, there are only three frequency shift components (density, elastic constant and wavelength).

Equation. The detailed calculation processes are as follows:
For a single-layer medium, the constitutive functions can be written as[2]

$$\gamma_{ik}\frac{\partial^2 u_j}{\partial x_i \partial x_k}+\frac{\partial \sigma_{ij}}{\partial x_i}=\rho\frac{\partial^2 u_j}{\partial t^2} \tag{1}$$

$$\frac{\partial D_i}{\partial x_i}=0 \tag{2}$$

$$\sigma_{ij} = c_{ijkl}\frac{\partial u_k}{\partial x_l} + e_{kij}\frac{\partial \varphi}{\partial x_k} \tag{3}$$

$$D_i = e_{ikl}\frac{\partial u_k}{\partial x_l} - \varepsilon_{ik}\frac{\partial \varphi}{\partial x_k} \tag{4}$$

where i, j, k = 1, 2, 3, represent the directions of x_1, x_2, x_3, which are corresponding to the coordinate system shown in Figure 3b in the paper. γ_{ik}, u_i, σ_{ji}, ρ are the external stress induced by bending, the mechanical displacement, the stress and the mass density, respectively. D_i, c_{ijkl}, e_{kij}, φ, ε_{ik} are the electric displacement, the elastic constant, the piezoelectric constant, the electric potential, and the dielectric constant, respectively.

Then the wave functions can be described as

$$\boldsymbol{u} = u(x_3)\xi,\ \boldsymbol{\varphi} = \varphi(x_3)\xi,\ \boldsymbol{\sigma}_3 = \sigma_3(x_3)\xi,\ \boldsymbol{D}_3 = D_3(x_3)\xi \tag{5}$$

where $\xi = \exp[j(\omega t - \boldsymbol{k}_1 x_1)]$, ω is the angular frequency, $\boldsymbol{k}_1$ represents the wave vector in the x_1 direction, $u(x_3) = [u_1(x_3)\ u_2(x_3)\ u_3(x_3)]^{\mathrm{T}}$ and $\sigma_3(x_3) = [\sigma_{13}(x_3)\ \sigma_{23}(x_3)\ \sigma_{33}(x_3)]^{\mathrm{T}}$. According to Eqs. (1) to (5), we can obtain the following equation:

$$\begin{bmatrix} \boldsymbol{Q}(z_t) \\ \boldsymbol{Q}(z_b) \end{bmatrix} = \boldsymbol{M}\begin{bmatrix} \boldsymbol{P}(z_t) \\ \boldsymbol{P}(z_b) \end{bmatrix} = \begin{bmatrix} \boldsymbol{M}_{11} & \boldsymbol{M}_{12} \\ \boldsymbol{M}_{21} & \boldsymbol{M}_{22} \end{bmatrix}\begin{bmatrix} \boldsymbol{P}(z_t) \\ \boldsymbol{P}(z_b) \end{bmatrix} \tag{6}$$

where z_t and z_b are the x_3 coordinates at the top and bottom surfaces, $\boldsymbol{P}(x_3) = [u(x_3)\ \varphi(x_3)]^{\mathrm{T}}$, $\boldsymbol{Q}(x_3) = [\sigma_3(x_3)\ D(x_3)]^{\mathrm{T}}$, and $\boldsymbol{M}$ is the stiffness matrix of the corresponding layer.

For multilayer media, the recurrence equation of $\boldsymbol{M}$ can be written as[1]

$$\boldsymbol{M}^N = \begin{bmatrix} \boldsymbol{M}_{11}^{N-1} + \boldsymbol{M}_{12}^{N-1}\left(\boldsymbol{M}_{11}^{n}\text{-}\boldsymbol{M}_{22}^{N-1}\right)^{-1}\boldsymbol{M}_{21}^{N-1} & \text{-}\boldsymbol{M}_{12}^{N-1}\left(\boldsymbol{M}_{11}^{n}\text{-}\boldsymbol{M}_{22}^{N-1}\right)^{-1}\boldsymbol{M}_{12}^{n} \\ \boldsymbol{M}_{21}^{n}\left(\boldsymbol{M}_{11}^{n}\text{-}\boldsymbol{M}_{22}^{N-1}\right)^{-1}\boldsymbol{M}_{21}^{N-1} & \boldsymbol{M}_{22}^{n}\text{-}\boldsymbol{M}_{21}^{n}\left(\boldsymbol{M}_{11}^{n}\boldsymbol{M}_{22}^{N-1}\right)^{-1}\boldsymbol{M}_{12}^{n} \end{bmatrix} \tag{7}$$

where $\boldsymbol{M}^N$, $\boldsymbol{M}^{N-1} = \begin{bmatrix} \boldsymbol{M}_{11}^{N-1} & \boldsymbol{M}_{12}^{N-1} \\ \boldsymbol{M}_{21}^{N-1} & \boldsymbol{M}_{22}^{N-1} \end{bmatrix}$, $\boldsymbol{M}^n = \begin{bmatrix} \boldsymbol{M}_{11}^{n-1} & \boldsymbol{M}_{12}^{n-1} \\ \boldsymbol{M}_{21}^{n-1} & \boldsymbol{M}_{22}^{n-1} \end{bmatrix}$ are the global stiffness matrix of total N layers, top N-1 layers and the nth layer, respectively.

The boundary conditions are given by[3,4]

$$\begin{aligned} &\lambda\big|_{x_3=Boundary} = 0,\ \sigma_3\big|_{x_3=Boundary} = 0,\ \text{free surface} \\ &\varphi\big|_{x_3=Boundary} = 0,\ \sigma_3\big|_{x_3=Boundary} = 0,\ \text{metallized surface} \end{aligned} \tag{8}$$

where λ is the surface charge density and φ is the electric potential.

By combining the boundary conditions and the stiffness matrix of total N layers, the resonant frequency as well as the acoustic wave amplitude on the SAW device surface can be solved.

Reference

[1] Rokhlin, S. I.; Wang, L. Stable Recursive Algorithm for Elastic Wave Propagation in Layered Anisotropic Media: Stiffness Matrix Method. *J. Acoust. Soc. Am.* **2002**, *112*, 822-834.

[2] Nalamwar, A. L.; Epstein, M. Surface Acoustic Waves in Strained Media. *J. Appl. Phys.* **1976**, *47*, 43-48.

[3] Xu, H.; Cao, Z.; Dong, S.; Chen, J.; Xuan, W.; Cheng, W.; Huang S.; Shi L.; Liu S.; Farooq U.; Qadir A.; Luo J. Flexible Dual-mode Surface Acoustic Wave Strain Sensor Based on Crystalline LiNbO3 Thin Film. *J. Micromech. Microeng.* **2018**, *29*, 025003.

[4] Zhang, Q.; Wang, Y.; Li, D.; Yang, X., Xie, J.; Fu, Y. Bending Behaviors of Flexible Acoustic Wave Devices Under Non-uniform Elasto-plastic Deformation. *Appl. Phys. Lett.* **2021** *118*, 121601.
